# Exploring the Role and Potential of Probiotics in the Field of Mental Health: Major Depressive Disorder

**DOI:** 10.3390/nu13051728

**Published:** 2021-05-20

**Authors:** Dinyadarshini Johnson, Sivakumar Thurairajasingam, Vengadesh Letchumanan, Kok-Gan Chan, Learn-Han Lee

**Affiliations:** 1 Novel Bacteria and Drug Discovery Research Group (NBDD), Microbiome and Bioresource Research Strength (MBRS), Jeffrey Cheah School of Medicine and Health Sciences, Monash University Malaysia, Bandar Sunway 47500, Malaysia; dinyadarshini.johnson2@monash.edu; 2 Clinical School Johor Bahru, Jeffrey Cheah School of Medicine and Health Sciences, Monash University Malaysia, Johor Bahru 80100, Malaysia; sivakumar.thurairajasingam@monash.edu; 3 Division of Genetics and Molecular Biology, Institute of Biological Sciences, Faculty of Science, University of Malaya, Kuala Lumpur 50603, Malaysia; 4 International Genome Centre, Jiangsu University, Zhenjiang 212013, China

**Keywords:** probiotic, microbiota, major depressive disorder, epigenetic, antidepressant

## Abstract

The field of probiotic has been exponentially expanding over the recent decades with a more therapeutic-centered research. Probiotics mediated microbiota modulation within the microbiota–gut–brain axis (MGBA) have been proven to be beneficial in various health domains through pre-clinical and clinical studies. In the context of mental health, although probiotic research is still in its infancy stage, the promising role and potential of probiotics in various mental disorders demonstrated via in-vivo and in-vitro studies have laid a strong foundation for translating preclinical models to humans. The exploration of the therapeutic role and potential of probiotics in major depressive disorder (MDD) is an extremely noteworthy field of research. The possible etio-pathological mechanisms of depression involving inflammation, neurotransmitters, the hypothalamic–pituitary–adrenal (HPA) axis and epigenetic mechanisms potentially benefit from probiotic intervention. Probiotics, both as an adjunct to antidepressants or a stand-alone intervention, have a beneficial role and potential in mitigating anti-depressive effects, and confers some advantages compared to conventional treatments of depression using anti-depressants.

## 1. Introduction

The foundation of a revolutionary medicine begins when the birth of theoretical knowledge is dynamically unified with its empirical application. The bidirectional communication within the microbiome–gut–brain axis (MGBA) is one such conceptualization and possibly a major breakthrough in existing medicine. The two-way interaction between the gut and the brain may not necessarily be an entirely alien concept previously, but the enormous attention this field of science has garnered in recent years is noteworthy. This school of thought dates back to around 400 BC when The Father of Medicine, Hippocrates, coined the idea “let food be thy medicine, and medicine be thy food” [1]. The bidirectional communication between the gut and the brain is mediated by trillions of microbes residing in the human gut through several mechanisms encompassing neural, immunologic and humoral pathways. This conceptualization has paved the way for scientists to explore and link various health conditions to the MGBA in an attempt to explain the possible etiopathologies involved in their occurrence. The number of studies involving some of the common disorders in the gastrointestinal, psychiatric, cardiometabolic and neurological domains, as well as cancers, in relation to the MGBA has been exponentially growing over recent decades and steadily moving towards a promising therapeutic direction in the context of human health [2,3,4,5,6,7,8].

The microbial pattern in every human is largely individualized and highly evolving across the lifespan. The first colonization of microbes in humans is thought to occur during birth although there are studies suggesting that this could occur as early as in utero via placental colonization [9,10,11,12]. Various factors influence the composition of microbiota in an individual which can be classified into extrinsic and intrinsic factors [2,6]. The influence of these factors largely manifests as the result of a dynamic and complex interplay between the environment and the human microbiome in a way yet to be fully understood. The emerging field of epigenetics provides one of the far-reaching theoretical explanations that describes the influence of environment on the human genome in the context of MGBA. The microbiota, which is a part of the environmental component, is capable of modifying the human genome expression in the gut–brain axis without altering the deoxyribonucleic acid (DNA) sequence through an epigenetic mechanism, thus resulting in changes which may possibly manifest as a health disorder. This epigenetic modification is long-lasting and heritable; however, it is reversible. Therefore, any disturbance in the intestinal microbial equilibrium, also referred as gut dysbiosis, induces a long-lasting yet reversible effect through the epigenetic modification of the human genome within the gut–brain axis pathway [13]. On the positive side, the reversibility of the microbial-induced changes in human biology allows scientists to explore various methods of microbiota modulation with the aim of reversing intestinal dysbiosis as part of a preventive and therapeutic approach [11,14].

Probiotics are among the most promising microbiota modulators that have led to a tremendous expansion of both animal and clinical studies over recent decades. The therapeutic potential of probiotics has been explored in a vast array of health conditions including type 2 diabetes, obesity, irritable bowel syndrome, asthma, cancers, arthritis, and mental health disorders [15,16,17,18,19,20,21,22]. The study of gut microbiome and microbial modulation using probiotics has extended to some of the most debilitating and severe mental disorders, including but not limited to major depressive disorder, bipolar disorder, and schizophrenia [23,24,25]. The immense potential of probiotics, particularly in treating mental disorders, has drawn a great deal of interest among researchers and clinicians. The number of research projects and clinical trials involving probiotics in the mental health domain has greatly accelerated within the last decade. There are indisputable advantages of probiotics over the conventional psychiatric treatment modalities, which highly favor its use in the treatment of psychiatric disorders. Clinical trials have demonstrated the efficacy of probiotic supplementation in ameliorating mental illness. The clinical efficacy of probiotics coupled with their lack of detrimental side effects and stigmatization make them a promising therapeutic intervention in mental disorders [26,27,28,29]. Although existing clinical trials are limited and more substantial research outcomes are needed, probiotics are a noteworthy therapeutic intervention as far as the mental health domain is concerned.

In this review, we aim to explore the role and potential of probiotics in the field of mental health with a particular focus on major depressive disorder (MDD). Recent meta-analyses and systematic reviews have substantiated the efficacy of probiotic supplementation in the treatment of depressive disorder in human subjects. Depression scores based on validated clinical scales and certain biomarkers associated with depression including cortisol, proinflammatory cytokines and brain-derived neurotrophic factor (BDNF) levels were used to demonstrate the anti-depressive effect of probiotic intervention [25,27,30,31]. Clinical trials of probiotics in patients with depression, although limited compared to pre-clinical studies, have demonstrated comparable outcome in terms of mediating anti-depressive effects, especially studies involving the measurement of biomarkers [18,32,33,34,35]. The basic understanding of the anti-depressive mechanism of probiotics has been largely gathered from animal studies [36,37,38,39,40,41,42]. Although research outcomes generally favor the utilization of probiotics in the treatment of depression, there are gaps when it comes to translating preclinical studies to human studies, due to the heterogenous nature of depressive disorder and the dynamicity of individual microbes [43,44]. Nevertheless, the exploration of MGBA in depressive disorder has certainly laid the foundation for the expansion of a clinical approach in the management of this debilitating disorder utilizing probiotics, despite the shortcomings, as this is an emerging, yet a rapidly growing field of research [23,43,45]. Therefore, we recognize the importance of understanding the basic mechanism of probiotics by specifically relating this to the possible etiopathologies of depression. Evidence is largely gathered from preclinical followed by clinical models. The integrative exploration of the mechanistic pathways of depression-associated etiopathology and probiotics in depression models is able to provide a more insightful and organized perspective in understanding the role and potential of probiotics in depression, while identifying possible gaps in the existing research knowledge. There are four domains which have been reviewed in this context including inflammation, neurotransmitters (serotonin (5-HT), dopamine (DA), noradrenaline (NE), gamma-aminobutyric acid (GABA)), the hypothalamic–pituitary–adrenal (HPA) axis and epigenetic mechanism. As healthcare professionals have an important intermediary role between the stakeholders of probiotics and consumers as the field of probiotics rapidly progresses in a therapeutic direction, we discuss probiotics from both scientific and clinical aspects to facilitate the practice of an evidence-based medicine, as well as to encourage the growth of this promising field.

## 2. Revisiting the Term “Probiotic”

The word probiotic is an etymological hybrid with both Latin (*pro*) and Greek (*bios*) origins which translate into “for life”. The use of fermented dairy products since time immemorial ascertains the deep-rooted existence and use of probiotics in human history. Over the years, there has been a widespread commercialization of probiotic-containing food products and probiotics as dietary supplements in the form of capsules, tablet, liquid and powder [46,47]. In 1905, a Russian scientist, Elie Metchnikoff, was the first to deduce the possible influence of microbes on human health by attributing the enhanced longevity of the rural Bulgarians to the regular consumption of fermented dairy products which contain *Lactobacilli* [48,49]. He considered *Lactobacilli* as probiotics and put forward the probiotic concept through his description of intestinal microbes which are dependent on food consumed, hence susceptible to compositional modifications. His hypothesis greatly contributed to the establishment and development of the modern dairy industry in France, the first in Europe [48]. The scientific community was first acquainted with the term probiotic in 1953 when a German scientist, Werner Kollath, referred to the active substances which are necessary for a healthy development of life as probiotics [48]. In 1989, Fuller provided a more specific definition of probiotics as live microorganisms which benefit the host animal by promoting intestinal microbial balance [50].

From the 1950s to the 1980s, probiotic research focused on identifying strains of probiotics and understanding the underlying mechanism of action. In 2000s, clinical trials using probiotics in human subjects started to emerge. However, the diverse range of probiotic strains used in various clinical trials made it difficult to derive disease-specific strains, as the therapeutic potential of probiotics is highly strain-dependent [49]. Over the years, the probiotic field continued to expand tremendously involving a huge array of probiotic stakeholders and consumers which, in a negative sense, gives rise to the exploitation and misuse of the term probiotic. This prompted scientists to revisit the concept of probiotics in 2013, when the International Scientific Association for Probiotics and Prebiotics (ISAPP) convened a panel of experts to examine and discuss the field. The widely adopted definition of probiotics published by the Food and Agriculture Organization of the United Nations and the World Health Organization (FAO/WHO) in 2001 was revisited and accepted with improved grammatical precision. A general consensus was reached by defining probiotics as “live microorganisms that, when administered in adequate amounts, confer a health benefit on the host” [51]. In the context of mental health, a more specific probiotic terminology known as psycho-biotics was introduced by Dinan et al. in 2013. Psycho-biotics are probiotics which exert a beneficial effect on patients suffering from mental health issues [52]. However, probiotics remains the preferred term due to the lack of independent criteria and mode of action of pyscho-biotics [47].

The core definition of probiotics has persistently remained central to the viability and active state of the ingestible substance; however, newer terminology such as parabiotic, postbiotic and para-psycho-biotic, which utilize an inactive, non-viable form of probiotics, are emerging. The health benefits of the inactivated form of probiotics and their metabolites are comparable to probiotics, and potentially a safer alternative to due to their downregulation of the host’s inflammatory response [47,53,54,55]. Another aspect of probiotic definition emphasizes the administration of an adequate amount to confer health benefits. Nonetheless, studies which demonstrate the dose–response relationship between probiotics and health outcomes in humans are insufficient [56]. There is no consensus on the adequate amount of probiotics which are required to elicit optimum health benefits specific to the target health condition of the host. However, a minimum amount of 10^9^ colony forming units (CFU) per daily dose of probiotic has been recommended to impart health benefits in general [46,57]. In the context of mental disorders, based on a systematic review, the doses of probiotics used were between 10^9^ and 10^10^ CFU over a duration of 2 weeks in animal studies and 4 weeks in clinical studies to demonstrate efficacy [58].

The ability of probiotics to confer health benefits to the host is the most cardinal element in the use of probiotic terminology. The diverse range of health benefits associated with the consumption of probiotics is certainly overwhelming. Ultimately, probiotics work by restoring intestinal dysbiosis and reversing associated adverse effects on gut health which have been identified as the culprit in the occurrence of various diseases. The anti-inflammatory, antipathogenic and antimicrobial properties of probiotics exert positive effects in terms of restoring and maintaining intestinal homeostasis and microbial balance. This contributes to the strengthening of bidirectional communication within the MGBA. The role of probiotics in immune modulation, gut homeostasis and restoration of microbial equilibrium confers both preventive and therapeutic health benefits to the host. Probiotics are, therefore, unapologetically perceived as the ‘fit-for-all’ formula which could potentially eradicate various disorders, especially those rooted in the gastrointestinal, immunologic, and neurological domains [42,59,60,61,62].

## 3. Mental Disorders: The Enigmatic Malady in the Field of Medicine

Mental disorders or mental illnesses are clinically diagnosable mental health problems with reference to the criteria outlined in the diagnostic manuals (i.e., Diagnostic and Statistical Manual of Mental Disorders, 5th Edition: DSM-5, International Classification of Diseases, Tenth Revision: ICD-10). Unlike other branches of medicine, the field of psychiatry often deals with ambiguity and clinical heterogeneity which impose major challenges in the management of psychiatric illnesses. There are no identifiable common pathogenic pathways and central disease mechanisms involved in the occurrence of mental disorders. The recognizable clinical patterns and symptoms of mental disorders are exemplified in the diagnostic manuals, which may be subjected to revision and modifications [63]. Whether psychiatric disorders can be classified as disorders of the brain or disorders that affect the brain remains a question. Mental disorders broadly involve mood, cognition, perception and behavioral aspects of the higher cortical function. However, it is impossible to localize the brain region involved and clinically measure the direct etiological factors which contribute to the development of a mental disorder. Mental disorders are, rather, a constellation of symptoms, often self-reported, which require the clinical expertise of a psychiatrist to arrive at a diagnosis [64,65].

Despite ambiguity and enigma, a new horizon and hypothetical perspective has begun to emerge in the field of mental health with the application of MGBA conceptualization. The idea of mental disorders merely contained within the head has been gradually experiencing a paradigm shift and a nudge to look beyond the neckline [64,66]. Landmark studies using germ-free mice models and fecal–microbial transplant (FMT) from patients with a particular mental disorder to healthy mice models have provided an insight into the implication of microbiomes in the development of neuropathological and behavioral symptoms which mimic the mental disorder that is being examined [67,68,69,70]. The consistent finding of gut dysbiosis or altered intestinal microbial composition and reduced diversity of microbial ecosystem within the gut among patients with mental disorders compared to corresponding healthy controls further ascertained the relationship between gut microbiome and its possible etio-pathological role in the occurrence of mental disorders [71,72,73,74].

Probiotics are involved in the modulation of microbiota within the MGBA. To explore the role and potential of probiotics, it is important to understand the target mechanisms in which probiotics exert their beneficial effect. The key point of interest will be in identifying some of the pathological mechanisms implicated in the occurrence of mental disorders and the possible mechanistic role of probiotics in tackling those pitfalls specific to each mental disorder.

## 4. Major Depressive Disorder (MDD)

MDD is clinically diagnosable when an individual is persistently in a depressed mood and/or experiencing anhedonia for at least 2 weeks along with other symptoms outlined in DSM-5, with a minimum five symptoms from: changes in appetite or weight, sleep disturbance, psychomotor changes, diminished concentration, fatigue, feeling of worthlessness, excessive guilty and suicidal ideation [75]. In this review, the term “depression” refers to the symptoms that depict MDD. Depression is one of the most common disorders affecting more than 350 million people worldwide with an estimated lifetime prevalence of up to 10.8% based on a community survey involving 30 countries between the years 1994 and 2014. The World Health Organization (WHO) predicts that depression may become the second leading burden of global disease, outranking cardiovascular disease, from its current fourth rank by 2030 [45,76]. An estimate of 800,000 deaths by suicide associated with depression yearly has been reported which reflects the debilitating and severe nature of this mental disorder [77].

The gut microbiome profile of depressive patients markedly differs from that of healthy controls, indicating the presence of significant gut dysbiosis in the former. At the phylum level, significant alterations within the main four phyla, *Bacteroidetes*, *Firmicutes*, *Proteobacteria*, and *Actinobacteria,* were reported in patients with MDD [72,78]. More specifically at the genus level, a notable decrease in *Bifidobacterium*, *Lactobacillus*, *Faecalibacterium*, *Ruminococcus* and an increase in *Prevotella*, *Clostridium*, *Klebsiella*, *Streptococcus* and *Oscillibacter* were observed in MDD patients [72,79,80]. These findings have prompted further hypothetical exploration of the possible etiopathology of depression from the microbiota–gut–brain angle.

## 5. Exploring the Role and Potential of Probiotics in Depression

The potential of probiotics in treating mental disorders has been explored using numerous models of a hypothetical mechanism, based largely on findings from studies performed in vitro and in vivo using animal models. The possible mechanistic role of probiotics in depression through their anti-inflammatory effects, restoration of gut permeability, modulation of neurotransmitters, attenuation of HPA axis and epigenetic mechanism will be discussed in more detail in the following subsections. Generally, the anti-inflammatory effects of probiotics have been demonstrated either through direct observation of reduced plasma concentration of proinflammatory cytokines or, indirectly, through suppression within the kynurenine pathway and restoration of gut permeability, which have been related to the etiopathology of depression [32,33,37,40,81,82,83,84]. The administration of probiotics has also been shown to restore and elevate the depleted levels of the neurotransmitters of interest, namely, 5-HT, DA, NE and GABA, which have been implicated in the occurrence of depression. This particular mechanistic attribute of the probiotic has been likened to the mechanism in which certain anti-depressants work, with comparable efficacy [36,85,86,87,88,89,90,91,92]. The role of probiotics in the attenuation of the exaggerated HPA-axis associated with depression has mainly been observed through the suppression of cortisol level, a stress biomarker in human subjects, cortisone level in animal models of depression [34,35,39,93,94,95], and alteration within the neurotransmitter circuitry involving the HPA-axis [87,96,97,98]. There are no current studies which have exclusively explored the epigenetic mechanism of probiotics in depression, nevertheless, the regulation of BDNF expression and histone deacetylase (HDAC) inhibition by butyrate produced by probiotics demonstrates the possible epigenetic potential of probiotics in the context of depression [58,99,100,101].

### 5.1. Inflammation

Inflammation affecting the central nervous system (CNS) through immune activation is one of the pathogenic mechanisms implicated in the occurrence of depression. Proinflammatory cytokines form the periphery and are the basis of neuroinflammation in depression, capable of disrupting the brain’s regulatory and signaling mechanisms involving behavioral and emotional aspects. Interleukin-6 (IL-6) and tumor necrosis factor-α (TNF-α) have been found to be significantly elevated in patients with MDD [102,103,104]. A compromised intestinal permeability which has been frequently associated with depression could be one of the contributing factors to elevated proinflammatory cytokine levels. This is consistent with the leaky-gut hypothesis, which narrates the vicious cycle of interaction between the gut microbiota, CNS and the periphery in the activation of inflammatory responses [103,105,106,107]. One of the proposed mechanisms in which proinflammatory cytokines induce depressive symptoms is through the activation of the indoleamine 2,3-dioxygenase (IDO) enzyme that facilitates the metabolic breakdown of tryptophan (TRP) into kynurenine (KYN) [108]. Elevated plasma KYN and the KYN/TRP ratio have been positively correlated with the severity of depressive symptoms. Increased plasma KYN has also been associated with suicidal behavior while decreased level has been associated with improved cognitive function in patients with MDD [32,109,110,111,112].

Probiotics mediate their anti-inflammatory effects via the modulation of proinflammatory cytokines, regulation of IDO activity and restoration of gut permeability. *Lactobacillus reuteri*, *Bifidobacterium infantis* and *Bifidobacterium adolescentis* are examples of probiotics which exert anti-inflammatory effects that could potentially ameliorate depressive symptoms [41,103]. The administration of *L. reuteri* improved depressive symptoms in mice models by restoring the *Lactobacillus* population and reversing stress-induced alteration of microbial hydrogen peroxide (H_2_O_2_), involved in the inhibition of IDO activity, and plasma KYN levels [82]. *L. reuteri* also mediates the anti-inflammatory effect by promoting the secretion of microbial histamine which suppresses the production of proinflammatory cytokines involved in IDO activation [81,83,113]. Mice treated with *B. infantis* were found to have reduced plasma concentration of TRP and suppressed IDO activity [40]. Both *B. infantis* and *B. adolescentis* have been associated with reduced plasma IL-6 concentration [37,40]. Probiotic *Lactobacillus plantarum* has demonstrated an anti-depressive effect in mice models and adults with depression [33,87] and is involved in the mitigation of inflammation through downregulation of IL-6 and TNF-α and significant restoration of intestinal permeability and *Lactobacillus* population [33,84]. In a randomized, placebo-controlled trial, the administration of *L. plantarum* significantly reduced plasma KYN level which has been correlated to improved cognitive function in patients with MDD [32]. *L. plantarum* also strengthens the gut barrier and modulates gut microbiota through the production of butyrate and butyrate-producing bacteria (i.e., *Lactobacillus*, *Bacteroidetes* and *Roseburia*) [84,87]. The probiotic *Faecalibacterium prausnitzii* is known to confer an anti-depressive effect through its ability to produce an abundance of butyrate and reduce IL-6 level [38]. Butyrate, a short-chain fatty acid (SCFA) produced by gut microbiota, confers a protective role on the gut permeability and exerts anti-inflammatory effects on various organs including the brain [114]. Butyrate has been demonstrated to ameliorate depressive symptoms and likened to an anti-depressant in mice models [58].

### 5.2. Neurotransmitters (Serotonin (5-HT), Dopamine (DA), Noradrenaline (NE), Gamma-Aminobutyric Acid (GABA))

The neurotransmitters 5-HT, DA, NE and GABA have been implicated in the pathophysiology of depression. These neurotransmitters, which are either gut or brain-derived, have imperative roles in the systemic homeostasis and regulation of neural circuits’ development and plasticity. Depletion in either of these neurotransmitters contributes to the development of various neuropsychiatric disorders including depression. Most antidepressants work by restoring the levels of monoamine neurotransmitters (i.e., 5-HT, DA and NE) [115,116,117,118]. The gut microbiota is involved in the modulation of neurotransmitters via either neural, humoral or immune-mediated pathways. Dysregulation and alteration in the gut microbiota have been found to impair the neurotransmitter circuitry [117,119]. The vagus nerve is one of the crucial mediators between the gut and the brain via the neural pathway, which is involved in the regulation of neurotransmitters by the gut microbiota [97,115].

Probiotics of the *Lactobacillus* and *Bifidobacterium* species are the most prominent neurotransmitter enhancers which mediate anti-depressive effect by producing various neurotransmitters including 5-HT, DA, NE and GABA [41,90]. The mechanism of some probiotics has been likened to that of antidepressants in the class of selective serotonin reuptake inhibitors (SSRI), which involve the 5-HT and brain-derived neurotrophic factor (BDNF). 5-HT and BDNF coregulate one another in mediating their physiological roles in the regulation of neural circuits’ development and plasticity. 5-HT stimulates BDNF expression while BDNF promotes the neurogenesis and neuronal survival of 5-HT. Impairment in the 5-HT–BDNF signaling mechanism has been implicated in the pathophysiology of depression [120,121]. The administration of *Lactobacillus helveticus* and citalopram, an antidepressant of the SSRI class, in rodent models demonstrated similar outcomes of increased levels of 5-HT with anti-depressive effects. The same study also demonstrated restored NE level, which has also been correlated to improved cognitive outcome in rodent models [85]. In another study involving mice models with corticosterone-induced depression, *Lactobacillus paracasei* demonstrated similar outcomes which were comparable to the efficacy of fluoxetine, an SSRI [36]. These probiotics upregulate the 5-HT-BDNF system to mediate the anti-depressive effect via involving different neural and immune-mediated humoral pathways within the MGBA [41]. In terms of NE, the *B. infantis* probiotic has been shown to elevate NE levels while downregulating IL-6 levels in depressed mice models [86]. *B. infantis* is capable of influencing the central NE system through the immune-mediated humoral route without involving the vagus nerve, unlike *L. helveticus* whose anti-depressive effect ceases with vagotomy [122,123]. DA plays a pivotal role in reward-related circuitry and its dysregulation has been associated with anhedonia, which is one of the cardinal symptoms of MDD [124]. The probiotic *L. plantarum* has been found to mediate the anti-depressive effect by increasing DA levels and downregulating monoamine oxidase A (MAO-A) expression in mice models [87,88]. The inhibition of MAO reduces degradation of DA and normalizes an exaggerated HPA-axis [87]. Selective inhibitors of MAO-A are known to be effective clinical antidepressants [125]. In terms of GABA, *Bifidobacterium* strains were ascertained as the most efficient GABA-producing gut-derived bacteria in a study involving 135 *Lactobacillus* and *Bifidobacterium* strains derived from the human gut [89]. In its sequential study, probiotics *Lactobacillus plantarum* and *Bifidobacterium adolescentis,* which were ascertained as the most efficient GABA-producers with anti-depressive potential, improved depressive behavior in mice models with an efficacy comparable to fluoxetine [90]. Studies have shown that the administration of *Lactobacillus brevis*, one of the main GABA-producers among *Lactobacillus* species, significantly improved insomnia in mice models and mediated anti-depressive effects, comparable to fluoxetine [89,91,92,116].

### 5.3. Hypothalamic–Pituitary–Adrenal (HPA) Axis

Stress is one of the major risk factors in the development of MDD. Chronic stress exposure has consequential effects on the gut microbial composition and HPA-axis [126,127]. Stress activates the HPA-axis which eventually stimulates the release of glucocorticoids (i.e., cortisol in humans and corticosterone in animals) from the adrenal cortex in response to stimulation by the adrenocorticotrophic hormone (ACTH) from the anterior pituitary gland. Increased circulatory glucocorticoids inhibit the hypothalamus secretion of corticotrophin-releasing hormone (CRH) and vasopressin, establishing a negative feedback circuit. On the other hand, stress-induced gut dysbiosis exacerbates gut inflammation and permeability, and stimulate the release of proinflammatory cytokines which further activate the HPA-axis. This vicious cycle of inflammation and HPA-axis activation involving the MGBA is prolonged by the persistent presence of stimulus in the form of chronic stress, rendering the immune system insensitive to the inhibitory signals of glucocorticoids and disrupting the negative feedback circuit. Persistently elevated circulatory glucocorticoids and proinflammatory cytokines also desensitize and downregulate the central neurotransmitter circuits and disrupt the inhibitory mechanism of the neurotransmitters, primarily GABA, on the CRH neurons of the hypothalamic paraventricular nucleus, rendering adverse effects on the brain neurotransmitter systems, although the exact mechanism via which neurotransmitters affect the HPA-axis remains unclear. An exaggerated HPA-axis response with a dysfunctional negative feedback system and increased inflammatory responses have been closely associated with the biological etiology of depression. This echoes the frequently reported elevated levels of cortisol, CRH and ACTH and proinflammatory cytokines in MDD patients [41,115,128,129,130,131].

Considering the multiple pitfalls within the MGBA involving the HPA-axis, probiotics have both direct and indirect roles in attenuating or normalizing HPA-axis hyperactivity through the restoration of gut dysbiosis and permeability and modulation of neurotransmitters [116]. The restoration of elevated cortisol levels is one of the main parameters of normalization of the HPA-axis. The administration of probiotic *L. rhamnosus* mitigated depressive behaviors in mice models by reducing stress-induced plasma corticosterone levels [39]. *L. rhamnosus* has been found to alter the expression of central GABA receptors and elevate GABA levels, which can be linked to its possible influence on the downregulation of the HPA-axis via the neural route (i.e., the vagus nerve) to exert an anti-depressive effect [97,98]. The administration of *L. plantarum* in depressed mice models showed reduction in MAO levels and increased levels of DA and its metabolites, with anti-depressive effects [87,96]. Excessive glucocorticoids hyper-stimulate enzymatic activity of MAO which is involved in the degradation of monoamine neurotransmitters; thus, it is proposed that *L. plantarum* regulates DA circuitry and the HPA-axis by modulating glucocorticoid-induced activation of MAO [41,132]. In mice models subjected to chronic stress, combination probiotics *L. Helveticus* and *Bifidobacterium longum* significantly improved depressive-like behaviors, reduced corticosterone levels, prevented stress-induced reduction of hippocampal neurogenesis of noradrenaline, and restored the gut barrier. Reduced cortisol levels have been linked to the attenuation of HPA-axis hyperactivity [95]. Consistently, in clinical trials of combination probiotics *L. Helveticus* and B. *longum*, clinical depression and mood outcomes were significantly improved in MDD patients and healthy human volunteers respectively, with reduced urinary cortisol levels at the end of probiotic trials [34,93]. In human subjects, the administration of *Lactobacillus casei* in stressed individuals reduced salivary cortisol levels and alleviated stress, as well as abdominal dysfunction [35].

### 5.4. Epigenetic Mechanism

Epigenetic mechanisms which involve the integration of environmental signals to modify gene expression, independent of changes in the actual DNA sequence, reveal another dimension of hypothetical understanding of the dynamic interaction between the gut microbiota and the host’s genome within the MGBA. Widely studied epigenetic mechanisms including DNA methylation, histone tail modifications and non-coding ribonucleic acids (RNAs) induce heritable changes in the host’s gene expression. The modifications in DNA and histone are enzyme-mediated which may either inhibit or promote gene expression [13,133,134]. One way in which the gut microbiota is postulated to regulate these epigenetic modifications is through its metabolites (SCFAs; butyrate, acetate, propionate), either via direct inhibition of the enzymes or alteration in the availability of substrates required for the enzymatic activity [135].

**Figure 1:**
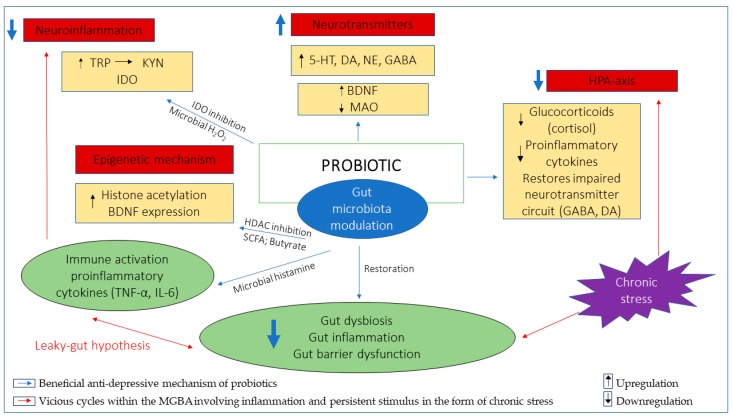
Illustration of potential anti-depressive mechanisms of probiotics, fundamentally involving gut microbiota modulation. The restoration of gut dysbiosis, inflammation and gut barrier dysfunction downregulates inflammation involving the TRP/KYN pathway which is implicated in depression and breaks the vicious cycles involving inflammation, the HPA-axis and persistent stimulus in the form of chronic stress. Probiotics promote synthesis of neurotransmitters either directly or indirectly by increasing BDNF levels and/or decreasing MAO levels to mediate anti-depressive effects. Probiotics attenuate exaggerated HPA-axis implicated in depression through downregulation of cortisol, proinflammatory cytokines and restoration of neurotransmitter circuits involving GABA and DA neurotransmitters. The epigenetic mechanism mainly involves butyrate-producing probiotics which inhibit HDAC and promote histone acetylation while upregulating BDNF expression. Tryptophan (TRP); Kynurenine (KYN); Indoleamine 2,3-dioxygenase (IDO); Serotonin (5-HT); Dopamine (DA); Noradrenaline (NE); Gamma-Aminobutyric acid (GABA); Brain-derived neurotrophic factor (BDNF); monoamine oxidase (MAO); Hydrogen peroxide (H2O2); Short-chain fatty acid (SCFA); Histone deacetylase (HDAC); Interleukin-6 (IL-6); Tumor necrosis factor-α (TNF-α).

In the context of the mental health domain, it is understood that the gut microbiota primarily exerts epigenetic effects within the MGBA involving key genes in the CNS through their metabolites (SCFAs, mainly butyrate) that ultimately influence behavioral outcomes [133]. Any significant gut dysbiosis negatively impacts epigenetic activity either directly or indirectly at the host genome level, which is known to be long-lasting, yet reversible. Probiotics are, therefore, utilized to modulate gut microbiota and restore epigenetic changes in a similar manner to that of the gut microbiota, which ultimately produces beneficial behavioral outcomes [133,136]. The regulation of BDNF expression and HDAC inhibition by butyrate are possible epigenetic mechanisms of probiotics in mediating anti-depressive effect. HDAC-mediated epigenetic alteration on histone tails downregulates histone acetylation and chromatin accessibility. The altered levels of histone acetylation and HDAC have been observed in the hippocampus of animal models of stress-induced depression [137,138,139]. Conversely, the administration of HDAC-inhibitors attenuated depressive-like behaviors and normalized epigenetic changes by promoting upregulation of histone acetylation [99,140,141,142,143]. Butyrate is one of the potent inhibitors of HDAC. The administration of butyrate has not only been shown to mitigate anti-depressive effects, but also promote hippocampal histone acetylation and BDNF expression as well as reducing gut inflammation [58,99,100,101]. Probiotics *L. plantarum*, *B. infantis*, *Clostridium butyricum and F. prausnitzii* are butyrate-producing probiotics which have been shown to ameliorate depressive behaviors in mice models [38,41,87,144,145]. *L. plantarum* and *B. infantis* also augment the production of butyrate-producing bacteria, while increased BDNF expression was observed with treatment with *B. infantis* and *C. butyricum* [87,144,145]. Treatment wjth *F. prausnitzii* in depressed mice models increased butyrate levels and reduced IL-6 levels, which have been associated with its ability to restore gut barrier function and mediate anti-inflammatory effects [38]. The anti-inflammatory effects of *F. prausnitzii* have been particularly attributed to the butyrate it produces, which downregulates proinflammatory cytokines by inhibiting HDAC1 in rats with induced colitis [146]. Although the epigenetic mechanism of probiotics seems indirect and remains largely unexplored, the butyrate-producing bacterial strains which target HDAC inhibition either at the hippocampal level or the intestinal epithelial, modulating BDNF expression and proinflammatory cytokines with anti-depressive effects, may serve as a suitable probiotic candidate to facilitate a better understanding of the epigenetic role and potential of probiotics in treating depression. An overview of the anti-depressive mechanisms of probiotics has been illustrated in Figure 1.

## 6. Discussion

The complex and heterogenous nature of a mental disorder with multiple plausible etiopathologies certainly imposes challenges from both scientific and clinical perspectives. The heterogeneity observed in mental disorders could possibly be attributed to the diverse and highly individualized microbial patterns across the human population [43]. The dynamic nature of the human microbiota profile which evolves across an individual’s life span due to various environmental and biological factors evokes questions pertaining to the potential of probiotics to offset such influences in exerting its long-term benefits [44]. Paradoxically, the same flexible nature of the human microbiota has catered to the development of therapeutic modulation using probiotics.

In the context of depression, the potential of probiotics has been explored by mechanistic relation to the possible etiopathologies associated with depression. Although, clinical studies are largely limited and discrepancies may arise when tested in human models, the available pre-clinical studies have produced substantial background to justify further expansion of probiotic research in human subjects. Inflammation, neurotransmitters, HPA-axis and epigenome involvement in the development of depression allows the utilization of probiotics to exert their therapeutic effect via modulation of gut microbiota within the MGBA. However, it is worth pinpointing that the causal link of depression to these possible pathologies is still under discussion and the heterogeneity of depressive symptoms could not possibly be explained using a single model or hypothetical mechanism [147]. Therefore, the multiple therapeutic effects produced by either a single probiotic strain (i.e., *B. infantis*, *L. helveticus*, *L. rhamnosus*) or a combination of *Lactobacillus* and *Bifidobacterium* probiotic strains may confer significant benefits in the treatment of depressive disorder [31,41]. The epigenetic mechanism gives a hopeful insight into the possible long-term probiotic-induced benefits; however, there is a dearth of studies available to arrive at a conclusion on the epigenetic potential of probiotics in depression.

Some of the challenges and concerns pertaining to the clinical management of depression are important aspects that cannot be overlooked in order to highlight the advantages of probiotics over the conventional treatment of depression. Clinically depressed patients are mostly managed in an outpatient setting, particularly those in the mild and moderate categories of MDD. Antidepressants are the most commonly prescribed class of drugs, with SSRIs as the first line of preferred antidepressants [148]. The mechanism via which antidepressants work has laid the foundation for the development of the monoamine hypothesis. For decades, the monoamine hypothesis, suggesting insufficiency within the neurotransmission circuit involving monoamine neurotransmitters, has predominantly ruled as the plausible mechanism in the occurrence of depression, hence the monopolization of antidepressants in its management [149]. However, antidepressants appear to be effective only in 46%–60% of patients [131]. Studies have found that patients who have shown improvement using antidepressants mainly resulted from placebo effects, and the antidepressant effects were unspecific. Further, the suicide rate and risks were similar between patients assigned antidepressants and placebo [150]. Tapering of anti-depressants is the norm via which antidepressants are discontinued [148]. However, the lack of guidelines on the discontinuation of antidepressants and the withdrawal effects associated with either abrupt or tapered discontinuation have been another concerning factor [151,152]. A systematic review of the withdrawal effects of antidepressants revealed that a weighted average of 56% of patients experience withdrawal effects with either abrupt or tapered discontinuation of antidepressants over varying durations from 6 weeks to several months. The side effects associated with prolonged use of antidepressants including weight gain, increased dependency, relapse rates and mortality risk, have also been highlighted as major concerns [152]. The use of SSRIs has also been associated with detrimental side effects in 46%–60% of patients, which include diminished sexual function, suicidality, apathy and addiction [153]. The stigma associated with the use of antidepressants also impacts adherence to the treatment [154].

Although most of pre-clinical studies and some clinical studies have demonstrated the efficacy of probiotics as a stand-alone treatment in depressive disorders, one of the recent meta-analyses of probiotic intervention in clinical depression concluded that probiotics are best used as an adjunct to antidepressants rather than as stand-alone treatment [25]. As an adjunct to antidepressants, probiotics could be utilized to tackle specific symptoms of depression. For example, GABA-producing probiotics have been associated with improved insomnia [155]. Probiotics *L. plantarum*, *L. paracasei*, *B. infantis* and *B. breve,* which are involved in the modulation of the DA system, may be effective in treating anhedonia [41]. In terms of duration of probiotic intervention, significant beneficial effects of probiotics in human subjects were observable after 4 weeks of administration [156,157]. Discontinuation of probiotic intervention requires no tapering and no withdrawal effects have been reported in any study to date. Most importantly, probiotics are generally safe with no harmful side effects reported thus far. Further, no stigma is associated with the use of probiotics and their consumption may also contribute to improvement in general health [26,157,158,159].

Last but not least, relating to the COVID-19 pandemic, the increased prevalence of depression in the community has become a huge concern in recent times. One of the recent meta-analyses of twelve large community-based studies published between January 2020 and May 2020 revealed a seven-fold increase in the pooled prevalence of depression amongst the general population during the COVID-19 outbreak, compared to the latest estimated global prevalence of depression based on Global Burden of Disease data in 2017 [160]. The impact of the pandemic on the mental health of the general population is immeasurable and imposes a huge burden on healthcare globally. In such circumstances, a non-prescription intervention such as probiotics becomes more relevant and valuable, especially for stress-induced depression [30,158].

## 7. Conclusions

It is beyond any reasonable doubt that probiotics have a beneficial role and potential in the mental health domain. The various mechanisms of probiotics overlap with the possible pathophysiological routes in depression, which may benefit from the probiotic modulation of microbiota within the MGBA. The mechanistic role and potential of probiotics, which are largely based on hypothetical connections based on findings from pre-clinical studies, require more intense research input based on human studies to justify the plausibility and validity of their proposed beneficial actions. Although probiotics appear to be a promising intervention in depression, the reproducibility of pre-clinical results in clinical studies remains a gap that needs to be addressed.
